# Disseminated Histoplasmosis Found in Bone Marrow in a Newly Diagnosed AIDS Patient: A Literature Review and Report of a Rare Case

**DOI:** 10.7759/cureus.35417

**Published:** 2023-02-24

**Authors:** Henrik Ghantarchyan, Yousuf Bholat, Amir Patel, Katherine Bourbeau, Dan Vo

**Affiliations:** 1 Internal Medicine, Arrowhead Regional Medical Center, Colton, USA; 2 Internal Medicine, St. George's University School of Medicine, St. George's, GRD

**Keywords:** bone marrow, disseminated disease, histoplasmosis, aids, hiv

## Abstract

Histoplasmosis is a rare fungal infection caused by the dimorphic species *Histoplasma (H.) capsulatum,* found in the Midwest and Central United States. Infection with *H. capsulatum *is observed in other regions beyond the Ohio and Mississippi River valley, including Mexico and Central and South America. There have been increasing reports of the disease occurring in Latin America in immunocompromised patients with human immunodeficiency virus (HIV). This case report details clinical findings of disseminated histoplasmosis in an immunocompromised patient, newly diagnosed with acquired immunodeficiency syndrome (AIDS) and initially presenting with sepsis of unclear source. The focus of this case report is the significance of detailed history-taking guiding for an appropriate investigation and recognition of the infectious source and giving insight into the management of disseminated histoplasmosis in the outpatient and inpatient settings.

## Introduction

Exposure to *Histoplasma* is commonly via moist environments that are left uninterrupted for prolonged periods of time such as caves, decaying buildings, and bridge foundations. Common animal vectors include birds and bats, and transmission is primarily through exposure to animal feces [1]. Fungal growth is primarily driven by the high nitrogen content of droppings. Fungal spores then become airborne if the soil is disrupted, which can be ingested by humans who come into contact with spores. Histoplasma initially infiltrates the respiratory system and usually does not cause disease in immunocompetent hosts but can cause moderate-severe pulmonary disease in patients who are immunocompromised [1]. Histoplasma is primarily endemic to the Ohio and Mississippi river valleys, with an incidence of 5%-20% [2,3].

Histoplasmosis is noted to be the most common endemic mycosis in the United States [2], but it is also noted to be the most prevalent systemic mycosis in Mexico [4]. Histoplasma is considered to be endemic in Mexico with cases found in 23 of the 32 states, and a higher number of reported cases are found in the states of Morelos, Guerrero, and Veracruz [4]. However, cases of Histoplasmosis are being identified in previously low endemicity areas in Mexico, which suggests more current information needs to be obtained regarding the geographic distribution of Histoplasma. The endemicity of Histoplasma is significant as a risk factor for developing a disseminated infection, as the population living in higher endemic areas has an increased rate of exposure, reportedly between 60% and 90% [4].

Signs and symptoms can emerge anywhere from three to 17 days and include fever, malaise, and productive cough with possible hemoptysis [1]. In rare cases, *Histoplasma* can disseminate into the blood and cause systemic symptoms such as nausea, vomiting, diarrhea, abdominal pain, skin ulcerations, and even shock. In terms of radiographic findings, calcified lung nodules, consolidation, and hilar lymphadenopathy may be seen in people living in endemic areas. The disseminated form of histoplasmosis can present with lung nodules in a miliary distribution [4]. Regarding laboratory abnormalities, lactate dehydrogenase (LDH) elevation, aspartate aminotransferase (AST), or alanine transaminase (ALT) alterations, decreased platelet, hemoglobin, or leukocyte counts are commonly encountered [4]. Although there are various clinically supportive data, isolation of *Histoplasma* in cultures provides definitive evidence for the infection, and a higher percentage of culture-positive cases can be found in disseminated histoplasmosis [4].

The following case presentation will detail the diagnostic workup that occurred for a patient newly diagnosed with AIDS and presenting with sepsis of unknown etiology, where information obtained from thorough history-taking proved vital as further diagnostic studies continued to be evaluated. Once the infectious etiology was identified, the management of the patient’s disseminated histoplasmosis required the initiation of an appropriate antifungal regimen followed by close outpatient follow-up.

## Case presentation

A 31-year-old male patient with a past medical history of anemia came into the emergency department (ED) with the chief complaint of progressively worsening abdominal pain, nausea, vomiting, and diarrhea for four to five days. Of note, the patient recently immigrated to Southern California from Central Mexico. Pertinent negative history review included no consumption of spoiled foods or travel outside of the region. He reported one prior sexual encounter with a single male partner one year before presentation and had never been tested for sexually transmitted infections (STI).

Initially, the patient was tachypneic (respiratory rate 24 per minute) and hypotensive (blood pressure 96/54), resulting in a qSOFA (quick sequential organ failure assessment) score of 2 on admission. On physical examination, oral thrush was seen in the tongue and oropharynx. There was no observed hepatomegaly or splenomegaly. Initial laboratory studies were significant for pancytopenia and elevated lactic acid (Table 1). Serology studies were confirmatory for HIV-1 infection. Blood and urine cultures were ordered, and he was started on empiric treatment with ceftriaxone 2g intravenous (IV) daily, metronidazole 500 mg every 8 hours, and fluconazole 800 mg oral daily. Subsequent blood and cerebrospinal fluid (CSF) cultures drawn three days after admission were unremarkable. Lab results later revealed positive for an EBV infection, a reduced CD4 count, and a urine antigen testing positive for histoplasmosis. At this time, he was continued on oral fluconazole. Antiretroviral therapy with bictegravir, emtricitabine, and tenofovir was started. His vital signs had normalized, and the patient was no longer septic.

**Table 1:** Significant lab results on presentation μL = microliter, g = gram, dL = deciliter, mEq = milliequivalent, mmol = millimole, mg = milligram, mL = milliliter

Blood Test Results	Patient Value	Reference Range
White blood cells (10*3 / µL)	2.4	4.5 - 11.1
Hemoglobin (g/dL)	9.9	13.0 - 17.0
Platelets (10*3/uL)	56	120 - 360
Lactate (mmol/L)	2.35	0.5 - 2
CD4 count (cells/uL)	41	490-1740
HIV-1 Viral Load RNA, PCR (copies / mL)	369,000	<20
EBV viral load PCR (copies / mL)	53,569	Not detected

Given his initial presentation of AIDS and the unclear etiology of laboratory derangements, multiple consultations were obtained, including Infectious Disease and Hematology. Due to the initial septic picture with an unclear etiology for pancytopenia, a bone marrow biopsy was pursued. Pathology was significant for hypercellular bone marrow with erythroid hypoplasia with atypical megakaryocytes. Bone marrow cultures were ordered; while awaiting results, trimethoprim-sulfamethoxazole (TMP-SMX) was subsequently added to his current regimen, however, it was discontinued as the patient had an allergic reaction, which included a diffuse, pruritic maculopapular rash. Instead, the patient was treated with clindamycin and primaquine for *Pneumocystis jirovecii (PJP) *prophylaxis. The patient was discharged home with antiretroviral therapy, clindamycin 450 mg oral four times daily, primaquine 52.6 mg oral daily, and fluconazole 800 mg oral daily with instructions for close outpatient follow-up.

**Figure 1:**
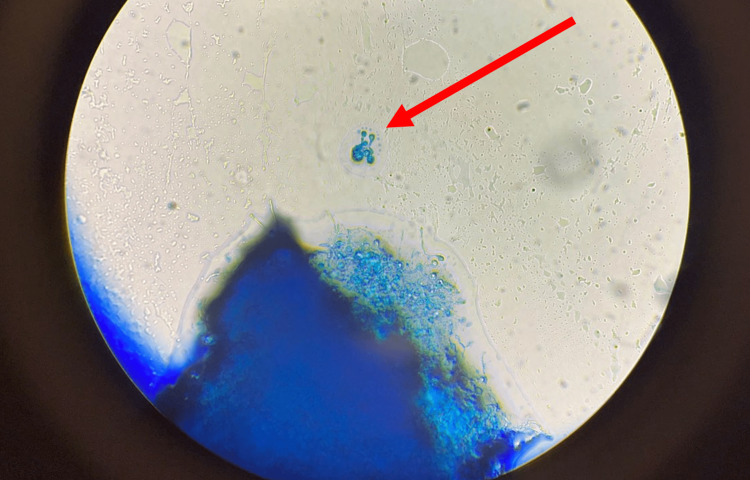
Histoplasma capsulatum (H. capsulatum) identified in bone marrow (red arrow)

**Figure 2:**
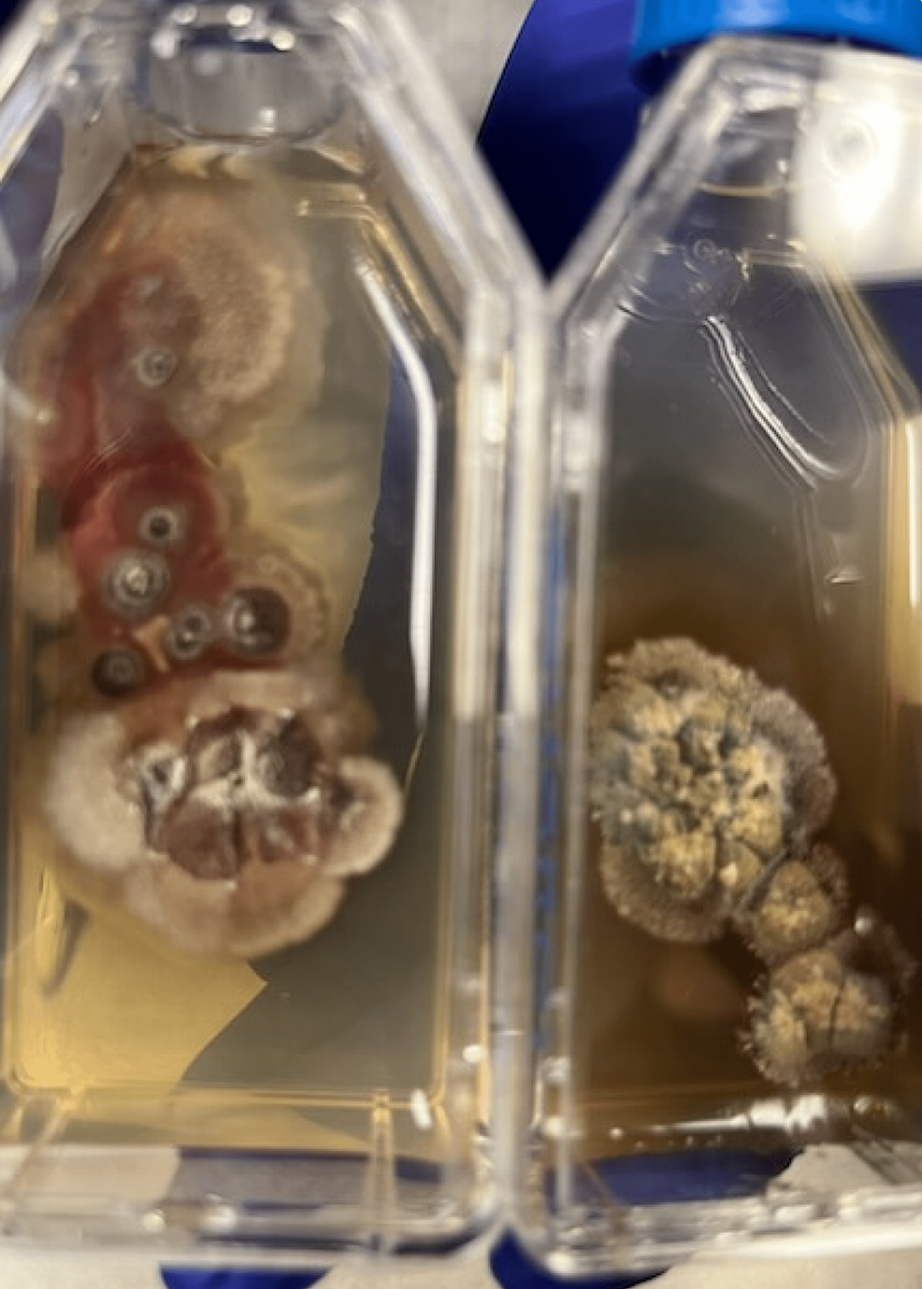
Bone marrow cultures positive for Histoplasma capsulatum (H. capsulatum)

The bone marrow biopsy revealed *Histoplasma capsulatum* (Figure 1). The bone marrow cultures also resulted positive for *Histoplasma capsulatum *(Figure 2). All other pertinent tests for STIs and opportunistic infections were negative for suspected infectious causes such as *Chlamydia trachomatis, Neisseria gonorrhoeae, Neisseria meningitidis, Toxoplasmosis, Coccidioides, *and *Cryptococcus neoformans*. The patient did follow up in the outpatient clinic setting with his primary care physician and Infectious Disease specialist. At this time, given severe histoplasmosis, the patient was offered amphotericin IV; however, he refused treatment, as he felt great on oral medications and preferred to continue the oral treatment. Treatment with oral fluconazole was changed to itraconazole with a loading dose of 200 mg three times a day for three days, then 200 mg daily thereafter. The patient was switched from clindamycin and primaquine for *PJP* prophylaxis to atovaquone 1500 mg oral daily. He was continued on his daily Biktarvy. 

**Figure 3:**
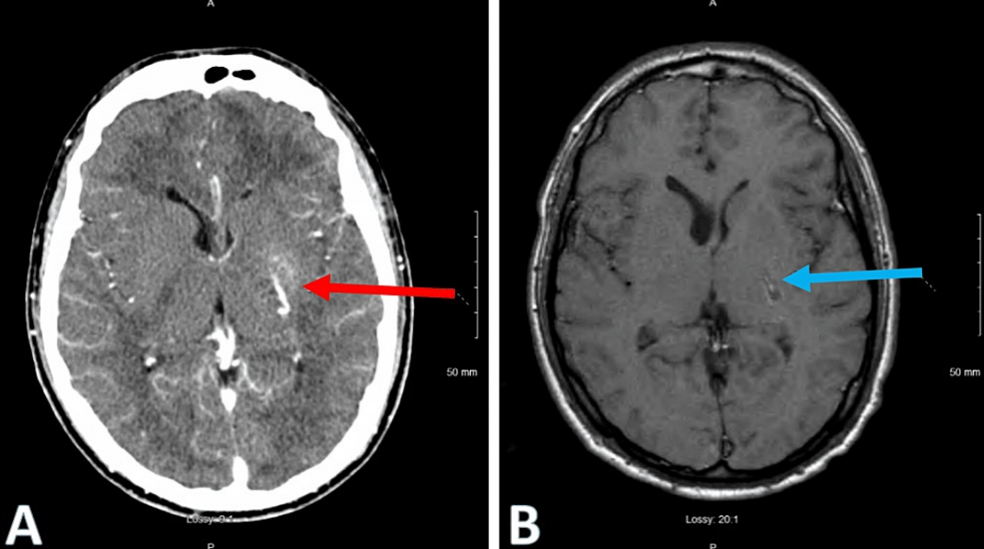
Computed tomography (CT) and magnetic resonance imaging (MRI) of the brain A. CT scan of the head with IV contrast suggestive of venous vascular malformation in the left basal ganglia (red arrow). B. MRI of the brain without IV contrast consistent with a 2 cm nidus of venous malformation in the left basal ganglia (blue arrow).

Approximately three months after discharge, the patient was readmitted for sepsis, with a new complaint of headache. Workup included a computed tomography (CT) scan of the head with contrast and magnetic resonance imaging (MRI) of the brain, which showed a 2 cm nidus of a venous vascular malformation in the left basal ganglia (Figure 3). Due to concern about neurologic spread, the patient was given an eight-day course of intravenous acyclovir and liposomal amphotericin B at 5 mg/kg for disseminated histoplasmosis. The patient continues to remain compliant with his Biktarvy, clindamycin, primaquine, and itraconazole treatment regimen.

At a six-month follow-up visit, an increase in his CD4 T-cell count with a decrease in his viral load was significant, which is shown in Table 2.

**Table 2:** Significant lab results at a six-month follow-up μL = microliter, g = gram, dL = deciliter, mEq = milliequivalent, mmol = millimole, mg = milligram, mL = milliliter

Blood Test Results	Patient Value	Reference Range
White blood cells (10*3 / µL)	3.8	4.5 - 11.1
CD4 count (cells/uL)	161	490-1740
HIV-1 Viral Load RNA, PCR (copies / mL)	109	<20

## Discussion

Disseminated Histoplasmosis in a newly diagnosed AIDS patient from a bone marrow aspirate is a rare case due to its presentation and aggressive course. There was no known travel or association with the geographic areas known to be endemic for histoplasmosis, as he recently immigrated from Mexico. However, it is important to consider the underlying factors that contribute to such an immunocompromised state, which in our case is AIDS, especially at the time of diagnosis. Other important factors to consider are age, sex, presentation type, CD4 count, symptoms, and imaging findings [3].

The initial presentation of histoplasmosis can be self-limited in healthy, immunocompetent individuals. Such symptoms commonly observed in acute pulmonary histoplasmosis range from asymptomatic to symptoms such as fever, headache, chest pain, and a dry cough [5]. Patients can also have chronic pulmonary histoplasmosis, leading to cavitations. Symptoms can be similar to chronic obstructive pulmonary disease (COPD), which include cough, sputum production, hemoptysis, and dyspnea [1]. When considering an immunocompromised population, a disseminated infection must always be considered, especially after correlation with a CD4 count. These symptoms include fever, malaise, anorexia, and weight loss [1]. This was similar to the symptoms that our patient presented with, which included nausea, vomiting, weight loss, diarrhea, abdominal pain, and skin ulcerations.

A retrospective study completed in Panama by Gutierrez et al. from January 1997 through December 2003 revealed the most common presenting symptomatology, diagnostic tests, and mortality rates. They looked at a total of 104 patients. The three most common symptoms were fever (seen in 96 patients), respiratory symptoms (seen in 66 patients), and weight loss (seen in 65 patients). A CD4 count of less than 100 cells/μL was seen in 73 patients. There were 12 patients that died. Out of the 104 patients, 100 were treated with IV amphotericin B, three were deceased without antifungal therapy, and only one was treated with itraconazole. There were only six patients that had a histoplasmosis relapse [3]. This report was very similar to our patient, as our patient declined IV amphotericin B when the disseminated disease was diagnosed and had a relapse in his disease.

As our patient initially had nonspecific symptoms, an appropriate workup revealed histoplasmosis in the urine. With an initial presentation of pancytopenia and a CD4 count of 41 cells/uL, there was increased suspicion of disseminated infection. The decision for a bone marrow biopsy was made by the treating team and consulting specialists.

When interpreting a bone marrow aspirate of an individual with HIV, clinicians should be aware of the common abnormalities to expect, which are marrow hypocellularity, myelodysplasia, dysmegakaryocytopoiesis, and dyserythropoiesis [6]. Interestingly enough, our patient was found to have hypercellular bone marrow with myeloid and megakaryocytic hyperplasia, and erythroid hypoplasia. When considering the characteristic finding of marrow hypoplasia, which was found in our patient, it is known to be infrequent and terminal in individuals diagnosed with AIDS [6].

A complete workup must include lab work and imaging. Appropriate lab studies that should be obtained include a complete blood count (CBC) to evaluate for bone marrow suppression, blood cultures, antibody tests, complement fixation, immunodiffusion, and enzyme immunoassays. These patients should also be tested for *Blastomyces dermatitidis *and *Coccidioidomycosis* as well [7]. Additionally, detection of the antigen is also important and useful in patients that don’t have a proper antibody response. Urinary antigen detection is found to have a higher sensitivity than serum antigen detection, 95% versus 86%, respectively [7].

Imaging is also important to obtain, initially with a chest X-ray, which can reveal granulomatous disease, especially in patients in endemic areas [7]. A computerized tomography (CT) scan or an MRI can also be obtained if there is a concern for a central nervous system (CNS) spread to the brain, which can show multiple lesions. An MRI would be helpful in identifying the classic “ring-enhancing” features of the lesion [8]. This can also be supported by a lumbar puncture, which would ultimately be both diagnostic and therapeutic. Findings of an isolated *Histoplasma capsulatum* from the CSF would be diagnostic. It is important to consider that cultures are insensitive, and due to prolonged growth times, this would result in treatment delays. A multicenter retrospective study looked into the sensitivity and specificity of both antigen and anti-Histoplasma antibodies using a new enzyme immunoassay, which revealed a sensitivity and specificity of 98% and 90.8%, respectively [8,9]. An MRI of the brain was obtained in our case, which showed a 2 cm nidus of venous vascular malformation in the left basal ganglia. However, CSF studies have been negative in our case on three separate occasions.

The treatment for disseminated histoplasmosis relies on the Infectious Diseases Society of America (IDSA) guidelines. Recommendations exist for moderately severe to severe progressive patients. The IDSA recommends one to two weeks of liposomal amphotericin B, 3 mg/kg per day followed by itraconazole 200 mg three times a day, then a yearlong course of itraconazole 200 mg twice a day [10].

## Conclusions

Histoplasmosis can be seen in immunocompromised patients in endemic areas such as Ohio and Mississippi River valley. We recommend that in symptomatic patients with newly diagnosed AIDS, disseminated disease of opportunistic infections be considered and worked up with a proper history taking, physical exam, lab work, imaging, and biopsies as necessary. Disseminated histoplasmosis requires appropriate, prompt treatment. In patients when CNS spread is considered, liposomal amphotericin followed by itraconazole should be initiated.
