# Correction: Disseminated Histoplasmosis Found in Bone Marrow in a Newly Diagnosed AIDS Patient: A Literature Review and Report of a Rare Case

**DOI:** 10.7759/cureus.c107

**Published:** 2023-04-07

**Authors:** Henrik Ghantarchyan, Yousuf Bholat, Amir Patel, Katherine Bourbeau, Dan Vo

**Affiliations:** 1 Internal Medicine, Arrowhead Regional Medical Center, Colton, USA; 2 Internal Medicine, St. George's University School of Medicine, St. George's, GRD

This article has been corrected at the request of the second author to change his affiliation from Arrowhead Regional Medical Center to St. George's University School of Medicine, St. George's, GRD.

The authors deeply regret that this error was not identified and addressed prior to publication.

